# Enhancement of the anomalous Hall effect by distorting the Kagome lattice in an antiferromagnetic material

**DOI:** 10.1073/pnas.2401970121

**Published:** 2024-07-15

**Authors:** Subhajit Roychowdhury, Kartik Samanta, Sukriti Singh, Walter Schnelle, Yang Zhang, Jonathan Noky, Maia G. Vergniory, Chandra Shekhar, Claudia Felser

**Affiliations:** ^a^Department of Topological Quantum Chemistry, Max Planck Institute for Chemical Physics of Solids, 01187 Dresden, Germany; ^b^Department of Chemistry, Indian Institute of Science Education and Research Bhopal, Bhopal 462066, India; ^c^Department of Physics and Astronomy, University of Tennessee, Knoxville, TN 37996; ^d^Min H. Kao Department of Electrical Engineering and Computer Science, University of Tennessee, Knoxville, TN 37996; ^e^Donostia International Physics Center, Donostia-San Sebastian 20018, Spain

**Keywords:** topology, anomalous Hall, distorted Kagome lattice, spin-ice

## Abstract

In topological magnetic materials, the topology of the electronic wave function is strongly coupled to the structure of the magnetic order. In general, ferromagnetic Weyl semimetals generate a strong anomalous Hall conductivity (AHC) due to a large Berry curvature that scales with their magnetization. In contrast, a comparatively small AHC is observed in noncollinear antiferromagnets. We investigated HoAgGe, an antiferromagnetic (AFM) Kagome spin-ice compound, which crystallizes in a hexagonal ZrNiAl-type structure in which Ho atoms are arranged in a distorted Kagome lattice, forming an intermetallic Kagome spin-ice state in the *ab*-plane. It exhibits a large topological Hall resistivity of ~1.6 µΩ-cm at 2.0 K in a field of ~3 T owing to the noncoplanar structure. Interestingly, a total AHC of 2,800 Ω^−1^ cm^−1^ is observed at ~45 K, i.e., 4 *T*_N_, which is quite unusual and goes beyond the normal expectation considering HoAgGe as an AFM Kagome spin-ice compound with a *T*_N_ of ~11 K. We demonstrate further that the AHC below *T*_N_ results from the nonvanishing Berry curvature generated by the formation of Weyl points under the influence of the external magnetic field, while the skew scattering led by Kagome spins dominates above the *T*_N_. These results offer a unique opportunity to study frustration in AFM Kagome lattice compounds.

## Significance

Distorting the ideal Kagome lattice by rotating the triangles in opposite directions breaks the inversion symmetry and results in a noncentrosymmetric structure. Consequently, the doubly degenerate Dirac cone of the Kagome lattice converts into a pair of Weyl points, which can generate large Berry curvature (BC)–induced anomalous Hall effects (AHEs). We find the magnetic Weyl semimetal HoAgGe with a distorted Kagome lattice, which exhibits the largest AHE among state-of-the-art antiferromagnetic systems, caused by nonvanishing BC generated by Weyl points formed by external magnetic fields. This study presents an example from the *R* AgGe series that span from nonmagnetic to magnetic compounds. Therefore, the distorted Kagome lattice offers a platform to achieve a range of topological quantum phenomena.

**Fig. 1.**
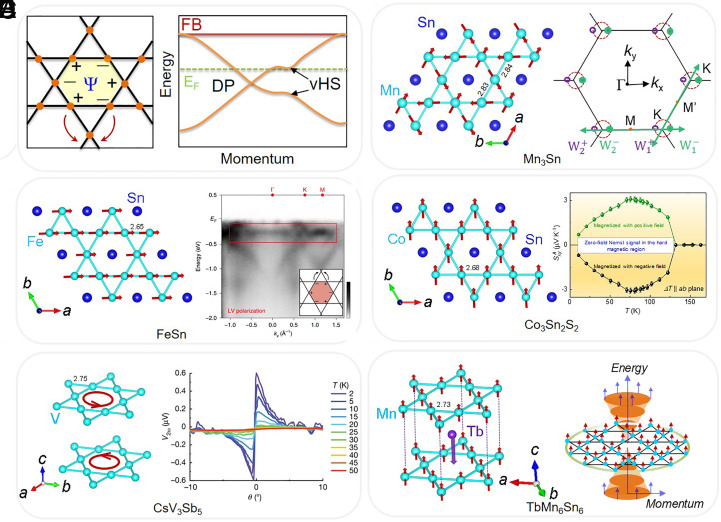
(*A*) *Left* panel: A general schematic for the confinement of an electron in the Kagome lattice in which the hopping of electrons within the corner-sharing triangular lattice is canceled by destructive interferences. “+” and “−” indicate the FB eigenstates of neighboring sublattices. *Right* panel: Model band structure corresponding to the lattice featuring Dirac point (DP), FB, and van Hove singularity (vHS). *E*_F_ represents Fermi level. (*B*) *Left* panel: Triangular spin structure of Mn_3_Sn in Kagome-plane. *Right* panel: Weyl point located around the *K* points of the Brillouin zone. (*C*) *Left* panel: Triangular spin structure of FeSn in Kagome-plane, *Right* panel: ARPES spectra of FeSn showing FB signature. Reprinted with permission from ref. 8, Copyright2019, Springer Nature. (*D*) *Left* panel: Triangular spin structure of Co_3_Sn_2_S_2_ in Kagome-plane, *Right* panel: Temperature-dependent zero-field Nernst thermopower. (*E*) *Left* panel: Spontaneous symmetry breaking crystal structure of CsV_3_Sb_5_, *Right* panel: Angular dependence of the electronic magnetochiral anisotropy at different temperatures (9). (*F*) *Left* panel: Triangular spin structure of TbMn_6_Sn_6_ in Kagome-plane, *Right* panel: Characteristics of a quantum-limit Chern magnet in the real space and the momentum space. All bond lengths are in Å.

In addition to the usual Bravais lattice, there are four other regular two-dimensional lattices of high symmetry—the square, triangular, honeycomb, and Kagome lattices—in which all vertices are equivalent to four, six, three, and four nearest neighbors, respectively. Such simple lattices capture modern exotic and crucial physical phenomena with promising applications in spintronics and quantum computing (1, 2). The Kagome lattice was first introduced by Syozi, which served as a rich system for exceptional quantum phases and fractionalized excitations in spin systems due to geometrical frustrations (3). Recently, however, this lattice has also been known to exhibit peculiar electronic properties such as flat bands (FBs), Dirac cones, van Hove singularities, and topologically nontrivial surface states (Fig. 1*A*) (4–8). FBs in momentum space result from strong electron localization in real space, which could induce nontrivial topological physics, such as in strongly correlated systems (6, 7). Therefore, Kagome materials are particularly well suited to study the interplay between magnetism, topology, and electron correlation.

The anomalous Hall effect (AHE), in which an electric current induces a transverse voltage proportional to the magnetization, is a widely studied phenomenon in modern condensed matter physics because of its applications in future low-power consumption and high-speed electronic devices (10). In ferromagnets, AHE can be observed at zero field, which is usually not observed in antiferromagnets (AFMs) (4, 10). However, Felser and coworkers theoretically modeled that an AHE can be observed in AFM Mn_3_Ge due to its noncollinear configurations giving rise to a finite Berry curvature (BC) contribution (11), in which magnetic Mn atoms are arranged in a Kagome-type lattice in the *ab*-plane (12). This prediction was later verified experimentally (Fig. 1*B*) (12, 13). FeSn has recently attracted considerable attention with their FBs, topological states, and itinerant magnetism derived from Kagome lattice structures (Fig. 1*C*) (7).

Our idea goes further, and through a combination of single-crystal growth, transport measurements, and theoretical calculations, a topological family of Shandite compounds with a Kagome lattice has been found in which the evidence for the Weyl semimetal (WSM) in Co_3_Sn_2_S_2_ has been observed (Fig. 1*D*) (4). Its topological electronic structure such as surface Fermi arcs, Weyl points, and linear bulk band dispersions over the Weyl points have been visualized using both angle-resolved photoemission spectroscopy (ARPES) and scanning tunneling microscopy (STM) (5, 14). In addition, it exhibits an intrinsic large anomalous Hall conductivity (AHC) with a giant anomalous Hall angle (AHA) and a large zero-field anomalous Nernst thermopower, which are the result of substantially enhanced BC arising from the Weyl nodes and the gapped nodal lines (4, 15). This is a remarkable compound that represents the rarest example in which AHC sustains up to 100 K, being the magnetic transition of 175 K. Moreover, the Hall conductivity per magnetic layer approaches its quantum value e^2^/h, where *e* is the elementary charge and *h* is the Planck constant.

After our milestone key findings, the search for Kagome compounds has escalated. A prototype structure in *A*V_3_Sb_5_ (*A* = K, Cs, and Rb) has emerged, where the two-dimensional vanadium Kagome network has attracted considerable attention (16–18). An anomaly at 94 K in the physical properties attributes to the formation of charge density waves (CDWs) (9, 19, 20). In addition to CDWs, superconductivity has also been observed, making this family an ideal model for investigating the correlation between superconductivity, CDWs, and nontrivial band topologies (Fig. 1*E*) (9). The CDW, which is achiral and putatively breaks the time-reversal symmetry, has been confirmed by various experiments, such as zero-field muon spin relaxation (µSR) and high-resolution magneto-optical Kerr effect (MOKE) measurements, but it is debatable (9, 21–23).

At the same time, a family of *RM*_6_*X*_6_ compounds known as “166” (*R* = heavy rare-earth element, *M* = transition metal, *X* = group IV element), which form a double Kagome lattice with *M* atoms, crystallize into the HfFe_6_Ge_6_-type structure (*P*_6_/*mmm*) and exhibit various quantum phenomena (Fig. 1*F*) (24–30). In terms of crystal structure, 166 compounds are stuffed versions of the centrosymmetric CoSn-type structure (known as B35) (27). In the CoSn-type structure, an alternative arrangement of Sn-centered Co Kagome lattices and Sn honeycomb lattices produces large hexagonal voids that act as hosts for cationic atoms. In the presence of a cation atom such as rare earths *R*, the Sn sites in the Kagome nets are pushed away from the center of the Co Kagome nets, thereby doubling the *c*-axis of the unit cell and making the two adjacent voids inaccessible. Various ordering patterns of *R*166 create a large structural diversity because *R* atoms can be placed at either 0 or 1/2 *z* coordinates. Importantly, unlike *A*V_3_Sb_5_, the “166” family has a greater degree of chemical tunability and a wide range of magnetic properties.

**Fig. 2.**
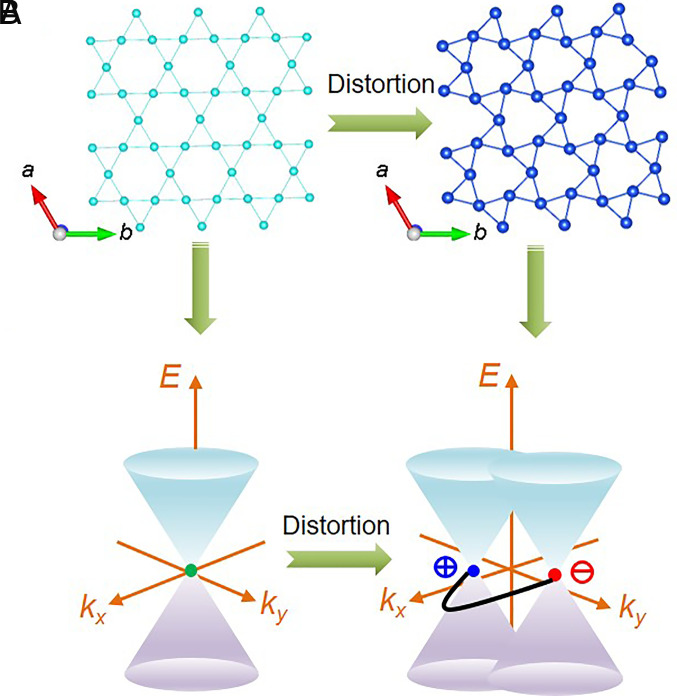
(*A*) *Left* panel: Standard Kagome lattice, *Right* panel: Distorted Kagome lattice. (*B*) Schematic representation of *Left* panel: Dirac cone in momentum space due to the Kagome lattice in real space. *Right* panel: Splitting of Dirac cone into Weyl cones due to distortion of the Kagome lattice.

In all the intensive research activities mentioned above, only the ideal Kagome structure has been considered. What is the next step in the Kagome lattice toward realizing exotic physics experimentally? In contrast to the standard Kagome lattice, distortions of this lattice have received little attention, despite being realized in some magnetic compounds and may have a much richer physical phenomenology. When two types of triangles in the standard Kagome lattice rotate in opposite directions, the rotation breaks the inversion symmetry, leading to a noncentrosymmetric structure. As a result, the doubly degenerate Dirac cone of the Kagome lattice transforms into a pair of Weyl points as sketched in Fig. 2.

**Fig. 3.**
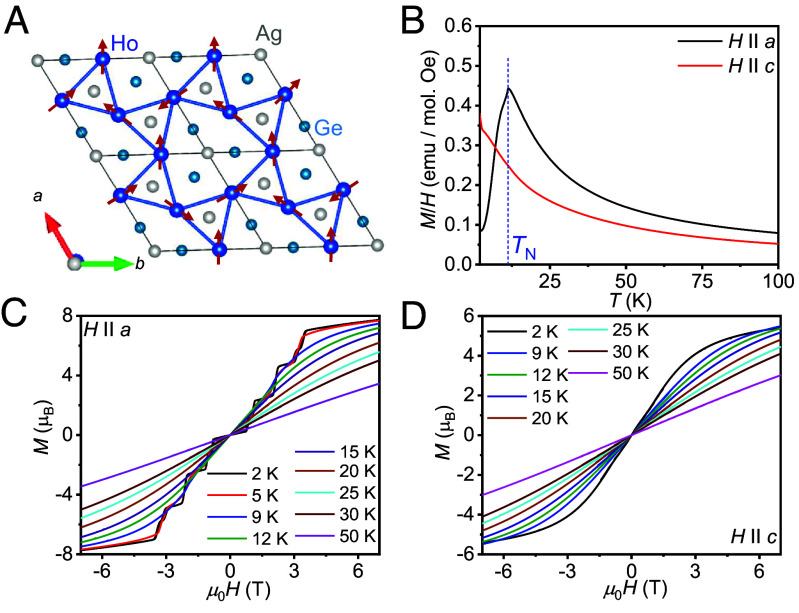
(*A*) Spin and crystal structures of HoAgGe, where the Ho atom forms a distorted Kagome lattice. (*B*) Temperature-dependent field-cooled magnetic susceptibility at *µ*_0_*H* = 5 mT for *H* || *a* and *H* || *c*. Isothermal magnetization for (*C*) *H* || *a* and (*D*) *H* || *c* at several temperatures.

Besides, exotic phases of matter can also form as a result of frustrated spin systems. One such phase of matter, called spin ice, is characterized by frustrated spins that obey local ice rules, analogous to the electric dipoles in water ice (31, 32). The corresponding material is the pyrochlore series, in which spin ice states are realized under the influence of a magnetic field (33, 34). In contrast, the intermetallic compound HoAgGe was recently found to be a spin ice system (35). HoAgGe crystallizes in a hexagonal AlNiZr-type structure (space group *P*-62*m*) in which the Ho atoms are triangularly coordinated with an interatomic distance of 3.68 Å. In this structure, the Ho atoms form a distorted Kagome lattice in the *ab*-plane (Fig. 3*A*). The substructure contains two types of triangles that rotate in opposite directions around the *c*-axis. The rotation breaks the inversion symmetry and results in a noncentrosymmetric structure. Such materials with localized spins in the Kagome lattice promote spin fluctuation (10) and can have significant consequences, especially in metals with itinerant electrons such as HoAgGe.

In the past, the noncoplanar spin texture of spin ice systems has been extensively explored and the value of the AHC has been found to be quite small (30 Ω^−1^ cm^−1^ for Pr_2_Ir_2_O_7_ and 20 Ω^−1^ cm^−1^ for Nd_2_Mo_2_O_7_) (36, 37). These AHCs are mostly extrinsic and arise from the skew scattering mechanism. However, various guidelines have been proposed so far to achieve a high AHE from the intrinsic contribution (38–40). Based on the hybridization strength (determined by the lattice constant) and the magnitude of the spin-orbit coupling (SOC) (determined by the atomic number), a strong correlation between the choice of materials and the desired bandgap is associated with a large AHE when the SOC-induced gap is small near the *E*_F_ (10). The presence of Weyl points and nodal lines in FM compounds leads to exhibit even large AHC from intrinsic BC contributions (4, 41). However, a comparatively small AHC is observed in noncollinear AFMs (8, 11–13). It is essential to search for more AFM systems with large AHC. Therefore, the search for new spin ice compounds with a high AHC is promising and desirable. Interestingly, HoAgGe meets the requirements of both AFM systems and spin ice states, providing an ideal platform to study the AHC.

In this study, we found that HoAgGe not only exhibits a large value of AHC below *T*_N_, but also shows the same order of magnitude at a temperature four times higher than *T*_N_, implying two different origins. Since the spin–orbit coupling in HoAgGe is quite large, the variation of the electronic response of HoAgGe with applied magnetic fields was investigated. A combination of electrical and magnetic measurements with theoretical calculations facilitated the observed large AHC. The studied single crystals were synthesized in an Ag–Ge flux (35).

## Results and Discussion

Fig. 3*B* shows the temperature variation of the field-cooled (FC) magnetic susceptibility χ(*T*) measured at *µ*_0_*H* = 5 mT for the *H* || *a* (*χ_a_*) and *H* || *c* (*χ_c_*) axes. The *χ_a_* spontaneously drops below *T*_N_ = 11.4 K, indicating a transition from a paramagnetic to an AFM-ordered state, which is consistent with the heat capacity (HC) measurement (*SI Appendix*, Fig. S3) (35, 42, 43). In contrast, the value of *χ_c_* increases with decreasing temperature.

To gain more insight into the magnetic phase diagram of HoAgGe, the isothermal magnetizations for the field *H* || *a* and *H* || *c* axes were measured (Fig. 3 *C* and *D*). At *T* = 2.0 K, the magnetization curve for *H* || *a* exhibits five plateaus with a small hysteresis. The previously reported metamagnetic transitions can be identified at *µ*_0_*H* ≈ 0.9, 1.1, 2.2, 3.1, and 3.4 T (35, 42–44). According to the existing neutron diffraction measurements on HoAgGe single crystals in magnetic fields, these phase transitions are associated with canted AFM states (35), which may have a strong impact on the electrical transport properties, as previously observed for canted AFMs Mn_3_Sn and Mn_3_Ge (11–13). In contrast, the magnetization curve for the *H* || *c* axis does not show any sudden jumps in the magnetic field. Therefore, the magnetization measurements indicate that the Ho spins in HoAgGe are more confined to the *ab*-plane. Our study now focuses on transport measurements for the *H* || *a* axis to understand the interplay between magnetism and topology in the system.

**Fig. 4.**
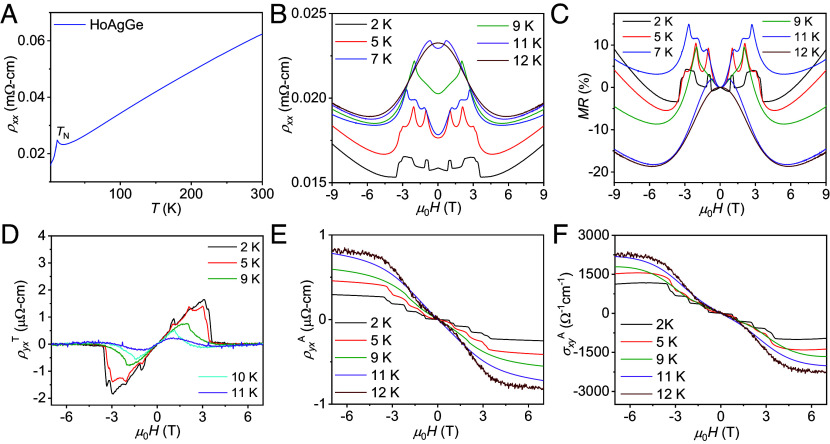
(*A*) Temperature-dependent resistivity, *ρ*_xx_. (*B*) Field-dependent *ρ*_xx_, (*C*) transverse magnetoresistance (MR), (*D*) topological Hall resistivity ($\rho_{\mathrm{yx}}^{T}$), (*E*) anomalous Hall resistivity ($\rho_{\mathrm{yx}}^{A}$), and (*F*) AHC ($\sigma_{\mathrm{xy}}^{A}$) at different temperatures. Measurement configuration: *H* || *a*-axis and *I* || *c*-axis of HoAgGe crystal.

At zero magnetic field, the longitudinal resistivity *ρ_xx_* for current along the *c*-axis of HoAgGe decreases completely linearly with decreasing temperature down to 20 K. This implies a crucial metallic nature of the compound with Planckian-type dissipation involved, which attracts attention for further study (45). The resistivity drops suddenly after exceeding the Néel temperature (*T*_N_ ~ 11.0(2) K) due to suppression of spin scattering. Typically, the value of *ρ_xx_* is 0.06 mΩ cm at 300 K and decreases to 0.015 mΩ cm at 2 K, resulting in a residual resistivity ratio [RRR = *ρ_xx_*(300 K)/*ρ_xx_*(2 K)] of 4, indicating a good crystal quality. The field-dependent *ρ_xx_* and the Hall resistivity, *ρ_yx_*, are significantly affected by the change of the magnetic property as shown in Figs. 4 and 6, and see *SI Appendix*, Figs. S4–S7. These two different behaviors can be classified into two regimes: 1) below *T*_N_ and 2) above *T*_N_.

The field dependence of the longitudinal and Hall resistivities of HoAgGe crystals (*T* = 2 to 12 K) is shown in Fig. 4. The field-dependent resistivity *ρ_xx_*(*H*) shows a complicated behavior at low temperatures (Fig. 4*B*). The *ρ*_xx_ initially shows a small decrease in the low-field regime (*μ*_0_H ≤ 0.50 T) Second, in an intermediate field range (0.50 T ≤ *μ*_0_*H* ≤ 3.5 T), several peaks in *ρ*_xx_ are observed due to increased spin-canting. Beyond the field range *μ*_0_*H* > 3.5 T, the spins are fully polarized as at higher temperatures. At 5 K, the *ρ*_xx_ increases slowly and then drops sharply at 1.0 T. Similar behavior is observed near ~2, ~3, and ~3.3 T, which cannot be described by standard MR models. However, these abrupt variations in *ρ_xx_* manifest at the critical fields of magnetic structure changes and correspond to the plateaus observed in Fig. 3*C*. These features appear only below 11 K due to spin–flip transitions and are more clearly visible in MR (MR = [*ρ*_xx_(*H*) − *ρ*_xx_(*0*)]/*ρ*_xx_(*0*)) (Fig. 4*C*). With increasing temperature, these peaks move to lower fields and eventually disappear above *T*_N_. Based on the magnetization data and a previous report (35, 44), these properties can be explained by in-plane spin flipping. The maximum negative value of the MR was observed to be ~20% at 12 K, close to the Néel temperature. The overall negative MR results from suppressed scattering due to spin disorder and the large positive MR is observed above 5 T due to the dominance of the Lorentz force (44). However, the negative MR is also a smoking gun experiment to prove transport in a Weyl system, but it is hard to confirm a solo contribution in such a strong spin scattering system.

**Fig. 5.**
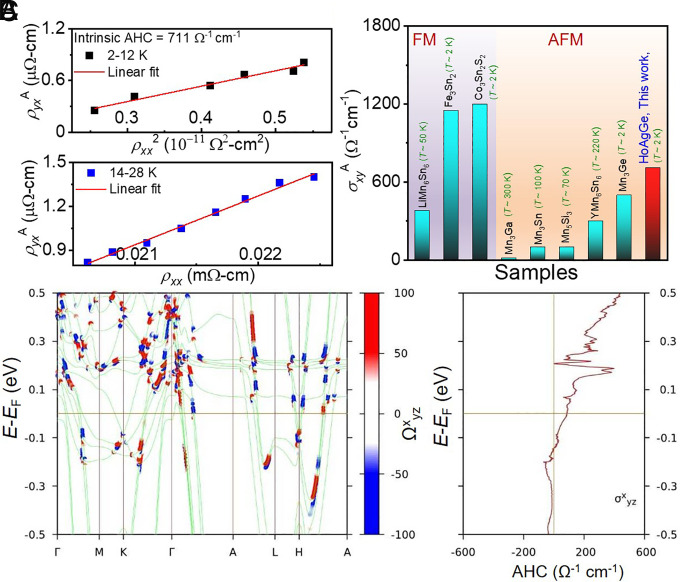
(*A*) *Upper* panel: Scaling relation between the anomalous Hall resistivity ($\rho_{\mathrm{yx}}^{A}$) to resistivity ($\rho_{\mathrm{xx}}^{2}$), confirming the intrinsic mechanism. *Lower* panel: Scaling relation between the anomalous Hall resistivity ($\rho_{\mathrm{yx}}^{A}$) to resistivity (*ρ*_xx_), confirming the skew-scattering mechanism. (*B*) Comparison of intrinsic AHC value ($\sigma_{\mathrm{xy}}^{A}$) of present system with previously reported ferromagnetic (FM) and AFM ordered compounds (4, 12, 13, 30, 51–54). (*C*) Band structure of HoAgGe for the FM state considering magnetic moment along the *a*-axis (*Left* panel) and theoretically calculated Hall conductivity as a function of chemical potential (*Right* panel). Red and blue colors indicate the BC contribution in the FM state.

**Fig. 6.**
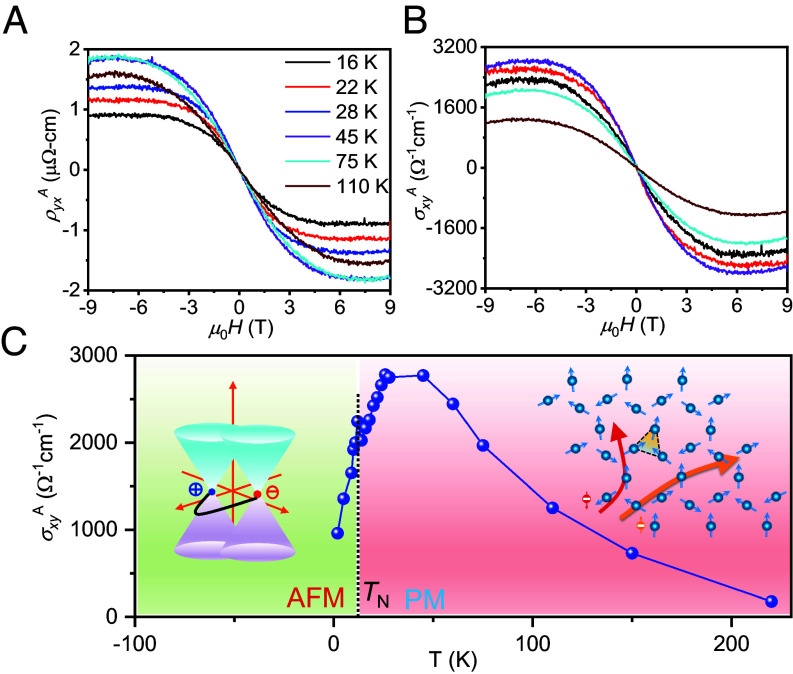
Field-dependent (*A*), anomalous Hall resistivity ($\rho_{\mathrm{yx}}^{A}$), (*B*) AHC ($\sigma_{\mathrm{xy}}^{A}$) at different temperatures. (*C*) Temperature-dependent AHC ($\sigma_{\mathrm{xy}}^{A}$) below and above the Néel temperature. AFM and PM stand for antiferromagnetic and paramagnetic, respectively. *Inset*: *Left* panel: Weyl points induced intrinsic AHC. *Right* panel: Spin cluster induced skew scattering mechanism. Measurement configuration: *H* || *a*- axis and *I* || *c*- axis of HoAgGe crystal.

In addition, magnetic ordering in metallic systems strongly influences the electronic band structure and its band topology. For example, the field-induced canted AFM structures of GdPtBi, Mn_3_Sn, and YbMnBi_2_ produce Weyl nodes, resulting in large AHE due to enhanced BC (11, 12, 46, 47) However, these AHE are typically lower compared to FM systems, such as Co_3_Sn_2_S_2_, Co_2_MnGa, and MnAlGe (4, 41, 48). We measured the field-dependent Hall isotherm *ρ_yx_*(*H*), which shows quite different behavior below and above *T*_N_ (Figs. 4–6). In general, the total Hall resistivity *ρ*_yx_ of a magnetic system has the following three contributions: (49) ordinary, anomalous, and topological Hall resistivities, i.e., *ρ*_yx_ = *R*_0_*H* + *R*_S_*M* + $\rho_{\mathrm{yx}}^{T}$. In our measured data below *T*_N_, an additional “hump-feature” is observed at fields *μ*_0_*H* < 3.4 T (Fig. 4*D*) after subtracting the first two terms, and such a hump is associated with either noncolinear or noncoplanar spin structures. Remarkably, the previous neutron scattering experiments in single crystals of HoAgGe reveal the evidence of noncoplanar structures in the applied magnetic fields (35). Due to the net spin chirality, the conduction electrons moving through noncoplanar structures accumulate a Berry phase, resulting in a topological Hall effect (THE). The maximum topological Hall resistivity of ~1.6 µΩ-cm was obtained at 2.0 K in a field of ~3 T (Fig. 4*D*). THE is unlikely to be large in AFM states with noncollinear spin structures. It appears that the electronic structure of HoAgGe and its magnetism are strongly related, allowing the observation of multiple topological states when a magnetic field is applied. There are several examples where the noncollinear spin structures induced by BC in the momentum space strongly couple the electronic and magnetic properties of MnBi_2_Te_4_ and MnBi_4_Te_7_ in the canted AFM state (49, 50).

The $\rho_{\mathrm{yx}}^{A}$ increases stepwise with the field, followed by saturation and a further increase. These steps are similar to the magnetization curve (Fig. 3*C*). The typical values of $\rho_{\mathrm{yx}}^{A}$ at 2.0 K and 12 K are ~0.3 μΩ cm and ~0.8 μΩ cm, respectively, and electrons are the majority charge carriers. Based on the anomalous Hall resistivity (Fig. 4*E*) and longitudinal resistivity (Fig. 4*B*), the total AHC was determined using the following equation: $\sigma_{\mathrm{xy}}^{A}$ ≈ $\rho_{\mathrm{yx}}^{A}$/$\rho_{\mathrm{xx}}^{2}$, when $\rho_{\mathrm{yx}}\ll \rho_{\mathrm{xx}}$. Fig. 4*F* shows the calculated $\sigma_{\mathrm{xy}}^{A}$ and the AHC values at 2.0 K and 12 K of 1,110 Ω^−1^ cm^−1^ and 2,250 Ω^−1^ cm^−1^, respectively. At 12 K, the AHA ($\sigma_{\mathrm{xy}}^{A}$/*σ*_xx_) was estimated to be about 4 %.

AHC typically measures the total contributions from various intrinsic and extrinsic mechanisms (10) The extrinsic contributions result from side jumps and skew scattering, while the intrinsic contributions are theoretically determined from electronic band structures (10). However, they can also be separated by using the empirical relationship: $\rho_{\mathrm{yx}}^{A}\left(T\right)={\alpha \rho }_{xx0}+{\beta \rho }_{xx0}^{2}+{\gamma \rho }_{\mathrm{xx}}^{2}\left(T\right)$, where *ρ_xx_*_0_ denotes the residual longitudinal resistivity (10). The first and second terms refer to the extrinsic contribution due to skew scattering and the side-jump mechanism, respectively, while the third term refers to the intrinsic contribution due to BC resulting in nontrivial bands. The intrinsic contribution to the AHC can be determined by plotting $\rho_{\mathrm{yx}}^{A}$ versus $\rho_{\mathrm{xx}}^{2}$ plot, where an intrinsic AHC of ~ 700 Ω^−1^ cm^–1^ is observed at temperatures below *T*_N_ (Fig. 5 *A*, *Upper* panel). Furthermore, the zero-field electrical conductivity of HoAgGe is of the order of ~10^4^ Ω^−1^ cm^–1^, which belongs to a good regime for intrinsic anomalous Hall transport. In general, FM systems exhibit a higher AHC than AFM systems, but a few magnetic WSM candidates have also shown large AHC, where BC contributions are enhanced. To date, the maximum observed AHC for AFM systems is 500 Ω^−1^ cm^−1^, about half the value for FM systems. However, HoAgGe exhibits an intrinsic AHC of ~700 Ω^−1^ cm^−1^ below *T*_N_, surpassing all known AFM systems and comparable to FM systems (Fig. 5*B*) (4, 12, 13, 30, 51–54). In contrast to Mn_3_Sn (12) and Co_3_Sn_2_S_2_ (4), HoAgGe does not exhibit remanent magnetization and behave as soft magnets without hysteresis. At a fixed temperature, there is a similar magnetic field dependence between the anomalous Hall contribution and the magnetization, indicating that the anomalous Hall contribution (proportional to *M*) dominates. In this case, it is possible to extrapolate the data on the *y*-axis in order to obtain the zero-field value, usually referred to as the field induced AHE. Similar results have been observed earlier in other magnetic Kagome systems such as LiMn_6_Sn_6_ (30), Fe_3_Sn_2_ (51), and YMn_6_Sn_6_ (54). To gain insight into the origin of the large AHC in AFM HoAgGe, detailed density functional theory (DFT) calculations were performed. Two magnetic states were considered in the electronic structure calculations, namely the FM (Fig. 5*C* and see *SI Appendix*, Fig. S8) and AFM (2 Ho-spin in – 1 Ho spin out and 1 Ho-spin in – 2 Ho spin out, spin-ice like configuration possible within the unit cell, cf. See *SI Appendix*, Figs. S9–S12) states. The FM configuration, with its Ho 4*f* spin aligned along the *a*-axis, was calculated including Hubbard *U* and SOC, along the high-symmetry line *Γ-M-K-Γ-A-L-H-A*. With GGA+*U*+SOC, we found a localized total magnetic moment of 9.68 *μ*_B_ (spin moment 3.64 *μ*_B_, orbital moment 6.04 *μ*_B_) at the Ho 4*f* site, which is consistent with the Ho^3+^ (4*f*^10^) charge state and the experimental measured value. Since the 4*f*-shell is more than half filled in Ho, the orbital magnetic moment is not quenched and is aligned parallel to the spin magnetic moment. Due to the large orbital magnetic moment at the Ho site, the effect of SOC in the electronic structure is found to be substantial.

In the electronic band structure, we observed several band crossings along the high-symmetry line *Γ-M-K-Γ-A-L-H-A*, within ~200 meV above and below the *E*_F_. It is noteworthy that a nonvanishing net BC is observed when SOC is combined with magnetism in HoAgGe to form AHC. To estimate the intrinsic contribution of the AHC near *E*_F_, a tight-binding Hamiltonian model based on Wannier functions which are maximally localized for Ho-*s*, *d*, *f*, Ag-*s*, *p*, and Ge-*p* was used. Considering the BC, the energy-dependent AHC of HoAgGe are calculated theoretically (Fig. 5*C*). The estimated value of the AHC is found to be in qualitative agreement with the experimentally observed value, indicating that the intrinsic BC causes the AHC in HoAgGe.

In addition to a large AHC below the *T*_N_, HoAgGe also exhibits a surprisingly large AHC above the *T*_N_, which is quite rare (Fig. 6). From Fig. 6*C*, the AHC initially increases and reaches maxima at 45 K, which is about 4 *T*_N_. It then decreases and maintains a finite value up to 200 K. The maximum $\rho_{\mathrm{yx}}^{A}$ value is approximately 2 μΩ cm at 45 K, which corresponds to a $\sigma_{\mathrm{xy}}^{A}$ value of around 2,800 Ω^−1^ cm^−1^ with an AHA of 7%. Unlike below *T*_N_, a relationship between the field-dependent Hall resistivities and magnetization is not expected, so the contribution of BC to AHC is not expected above *T*_N_. It seems that the noncoplanar fluctuations can potentially exist above the magnetic ordering temperature (55), which strongly enhances the spin-cluster skew scattering (56). In the present case, the spin-cluster AHC is maximized at a finite temperature (~45 K) in the presence of a constant magnetic field, as shown in Fig. 6*B*.

For such a large AHC, the skew scattering plays a crucial role, especially in the noncoplanar spin lattices, e.g., spin cluster (57), spin chiral (56), and triangular spin (37). In the spin structure of HoAgGe, when an external field causes magnetic fluctuations to act as scattering centers, the distortion of the local order within a triangular spin cluster or a tiled cluster in a Kagome lattice produces an enhanced skew scattering potential above the *T*_N_ (Fig. 6 *C*, *Rightpanel*). More precisely, spin cluster skew scattering contributes to the AHC by averaging the spin chirality 〈*S*_i_ ⋅ (*S*_j_ × *S*_k_)〉 rather than the average chirality 〈*S*_i_〉 ⋅ (〈*S*_j_〉 × 〈*S*_k_〉), similar to the intrinsic mechanism (57). This scenario is also supported by the large orbital magnetic moment of Ho atoms due to SOC, which provides the main centers of skew scattering. The large AHC in HoAgGe over *T*_N_ is similar to that in KV_3_Sb_5_ and EuAs. The former one is a Kagome metal without magnetic order (18) and the latter has a large spin fluctuation in the triangular lattice with large AHC (38). Furthermore, it is evident that the local spin chirality in HoAgGe is gradually reduced by thermal fluctuations above *T*_N_, which may lead to larger AHC in frustrated Kagome systems with finite spin chirality. In view of these results, future theoretical studies may unravel the details of the scaling relations.

In summary, the magnetoelectric transport of AFM HoAgGe containing a distorted Kagome lattice of Ho atoms has been studied. HoAgGe exhibits a large intrinsic AHC of ~700 Ω^−1^ cm^−1^ at 11 K close to the Néel temperature, and the AHC value is higher than that of state-of-the-art AFM systems. This large value can be attributed to the enhanced BC and the density of states corresponding to Weyl points close to the Fermi level due to TRS breaking rather than inversion symmetry breaking. Moreover, the large extrinsic AHC of ~2,800 Ω^−1^ cm^−1^ and a corresponding AHA of 7% were observed at 45 K due to a spin-chirality-induced skew scattering mechanism, and the temperature is much larger than the AFM ordering temperature. In addition, the large topological Hall resistivity of ~1.6 µΩ-cm was observed at 2 K, arising from the noncollinear magnetic structure of the material. From these results, we can relay that the AFMs with Kagome spin ice state have the potential for even larger AHC, which warrants further investigation to other family members of Kagome magnets in *R*AgGe compounds in terms of magnetic skyrmions, as well as magnetoelectrical and thermal transport studies. Our present results are relatively simple but realistic distortion of the Kagome lattice is capable of causing a diverse set of interesting and unexpected magnetic and quantum phenomena. ZrNiAl-type intermetallic compounds with space group *P-62m* are an intriguing class of distorted Kagome lattices, ranging from nonmagnetic to magnetic materials, which may host additional exotic topological physics interplaying between topology, electron correlation, and magnetism.

## Note Added

During the review process of the manuscript, a similar work with AHE in HoAgGe was found (58).

## Materials and Methods

### Single-Crystal Growth of HoAgGe and Characterizations.

Single crystals of HoAgGe were grown from an Ag–Ge rich melt (*SI Appendix*, Figs. S1 and S2). As-purchased elemental Ho (alfa aesar, 99.9%), Ag (alfa aesar, 99.999%), and Ge (chemPur, 99.9999+%) pieces were mixed in the molar ratio Ho: Ag: Ge: of 0.06:0.705:0.235 in an Argon-filled glove box. All of the elements were loaded into an alumina crucible which was vacuum sealed in a quartz tube under a pressure *p* of ~10^−5^ mbar. The tube was heated to 1,150 °C for 10 h, then held for 10 h before being progressively cooled to 836 °C for 76 h. After centrifugation at 836 °C to remove excess flux, the crystals were recovered. White-beam backscattering Laue X-ray diffraction was used to determine the single crystallinity of the as-grown crystal. Scanning electron microscopy with an EDAX analyzer was used to evaluate the composition of the HoAgGe crystals. The typical size of the crystals is 3 × 0.3 × 0.3 mm^3^.

### Electrical Transport and Magnetization Measurements.

An MPMS3 magnetometer (Quantum Design) was used to measure the magnetization. An integrated magnetic cryostat measurement system (PPMS9, Quantum Design) was used for further low-temperature measurements. Using the heat capacity (HC) option of the PPMS9, the HC, *C*_p_, of the sample was measured. The electrical transport properties of the PPMS9 were measured using the electrical transport option (ETO) of the instrument. A rectangular bar was cut from the sample, and six 25-μm Pt wires were attached with the silver paint to measure the longitudinal and Hall resistivities. To eliminate the effect of contact misalignment, the field-dependent longitudinal and Hall resistivities data were symmetrized.

### Computational Methodology.

The electronic structure calculations were performed based on the DFT using the plane-wave projected augmented wave (PAW) method as implemented in Vienna ab initio Simulation Package (VASP) (59–61). A plane-wave cutoff of 500 eV and a *k*-points mesh of 7 × 7 × 9 was used for the self-consistent calculations. We used the Perdew–Burke–Ernzerhof (PBE) (62) exchange-correlation functional within the generalized gradient approximation (GGA). To treat the strongly correlated Ho 4*f* electrons, we used the DFT+*U* method (63), where an onsite Coulomb interaction parametrized with a Hubbard *U* = 4.0 eV. We constructed a maximumly localized Wannier functions (MLWF) Hamiltonian from the GGA+*U*+SOC Bloch wave functions using the Wannier90 package (64–67).

Atomic orbital-like MLWFs of Ho*-s,d,f,* Ag*-s,p,* and Ge*-p* states are considered to construct the tight-binding Hamiltonian, which reproduces the spectrum of the system accurately in the energy window of ±2 eV around the Fermi energy. With the tight-binding model Hamiltonian, we calculated the BC $\overset{\vec{\Omega_{n}}}{\;}$ using the linear response Kubo formula approach as follows:

[1] $${\text{Ω}}_{n,ij}=\sum_{m\neq n}\frac{\langle n|\frac{\partial H}{\partial k_{i}}|m\rangle \langle m|\frac{\partial H}{\partial k_{j}}|n\rangle -\left(i↔j\right)}{{\left(\varepsilon_{n}-\varepsilon_{m}\right)}^{2}},$$

where *m* and *n* are the eigenstates and *ε* are the eigen energies of the Hamiltonian *H.*

Subsequently, AHC $\sigma_{\mathrm{xy}}^{A}$ was calculated using the equation (67):

[2] $$\sigma_{\mathrm{xy}}^{A}=\frac{-e^{2}}{h}\sum_{n}\int \frac{\mathrm{dk}}{{\left(2\pi \right)}^{3}}\Omega_{n,xy}\left(k\right)f_{\mathrm{nk}},$$

where $f_{\mathrm{nk}}$ is the Fermi–Dirac distribution function with band index *n* and wave vector *k*.

We used a very dense *k*-point mesh of 300 × 300 × 300 to realize the integration over the whole Brillouin zone for the calculation of AHC using Eq. **2**.

## Supplementary Material

Appendix 01 (PDF)

## Data Availability

All study data are included in the article and/or *SI Appendix*.
