# Supplementary material for: Enhancement of the anomalous Hall effect by distorting the Kagome lattice in an antiferromagnetic material
Source: Proc Natl Acad Sci U S A. 2024 Jul 15;121(30):e2401970121. doi: 10.1073/pnas.2401970121 (PMC11287124; doi:10.1073/pnas.2401970121)
Supplement: Supplementary file 1 — Appendix 01 (PDF) [file pnas.2401970121.sapp.pdf]

PNAS

Supporting Information for

Enhancement in the Anomalous Hall Effect by Distorting the Kagome Lattice in an Antiferromagnetic Material

Subhajit Roychowdhury[a,b,1] Kartik Samanta[a], Sukriti Singh[a], Walter Schnelle[a], Yang Zhang[c,d], Jonathan Noky[a], Maia G. Vergniory[a,e], Chandra Shekhar[a], and Claudia Felser[a,1]

[a]Max Planck Institute for Chemical Physics of Solids, 01187 Dresden, Germany; [b]Department of Chemistry, Indian Institute of Science Education and Research Bhopal, Bhopal-462 066, India; [c]Department of Physics and Astronomy, University of Tennessee, Knoxville, Tennessee 37996, USA; [d]Min H. Kao Department of Electrical Engineering and Computer Science, University of Tennessee, Knoxville, Tennessee 37996, USA; [e]Donostia International Physics Center, 20018 Donostia-San Sebastian, Spain

[1]To whom correspondence may be addressed.

Email: Subhajit.Roychowdhury@cpfs.mpg.de; Claudia.Felser@cpfs.mpg.de

This PDF file includes:

Figures S1 to S12

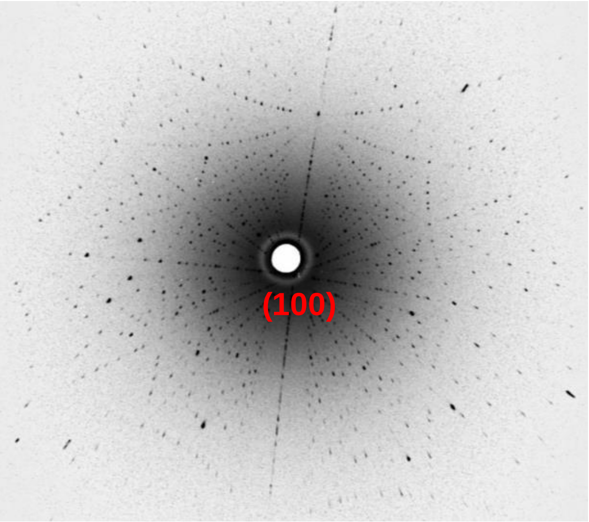

Fig. S1. Laue X-ray diffraction pattern of the [100] direction-oriented HoAgGe crystal.

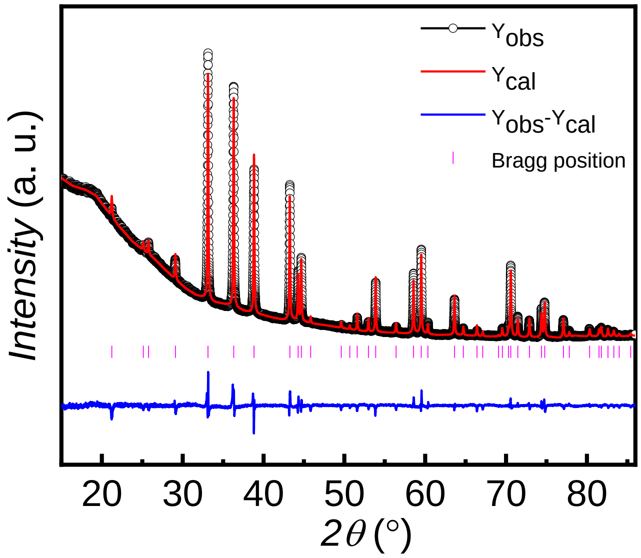

Fig. S2. Pattern matching of XRD data from ground single crystal powder confirms the phase purity of HoAgGe crystal.

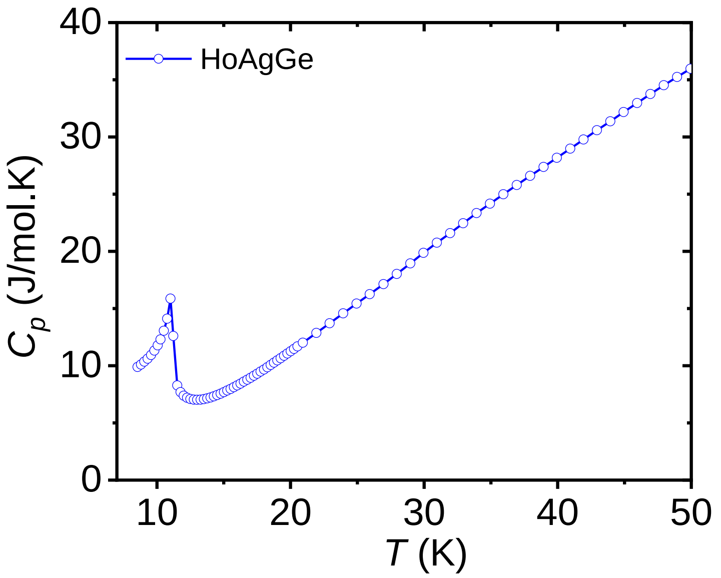

Fig. S3. Heat capacity of HoAgGe.

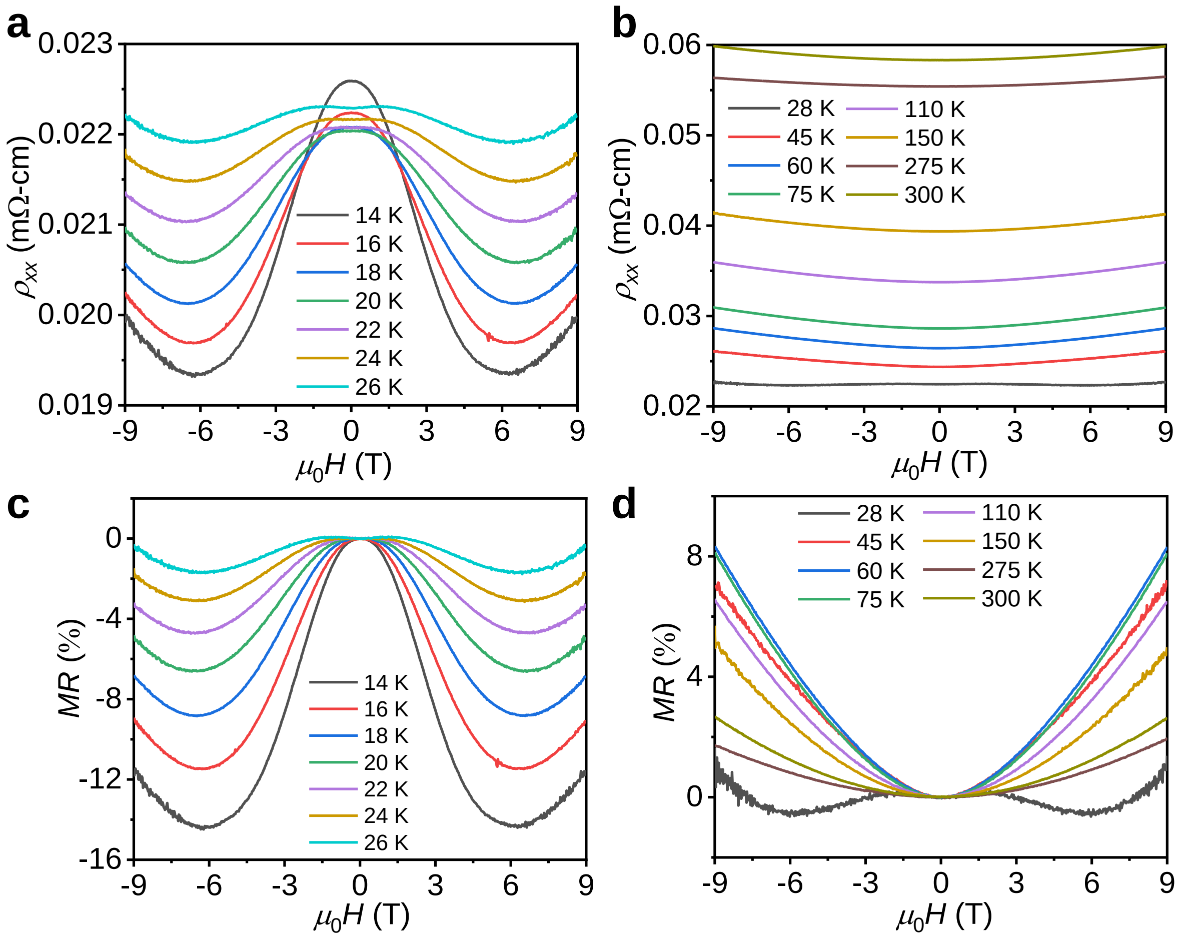

Fig. S4. Field dependent **a-b**. resistivity, ρ_{xx} and **c-d**. transverse magnetoresistance (MR) of HoAgGe at different temperatures. Measurement configuration: $H \parallel a$ and $I \parallel c$ axis of HoAgGe crystal. In the main text, electrical transport data below and above (few temperatures) the Néel temperature are presented.

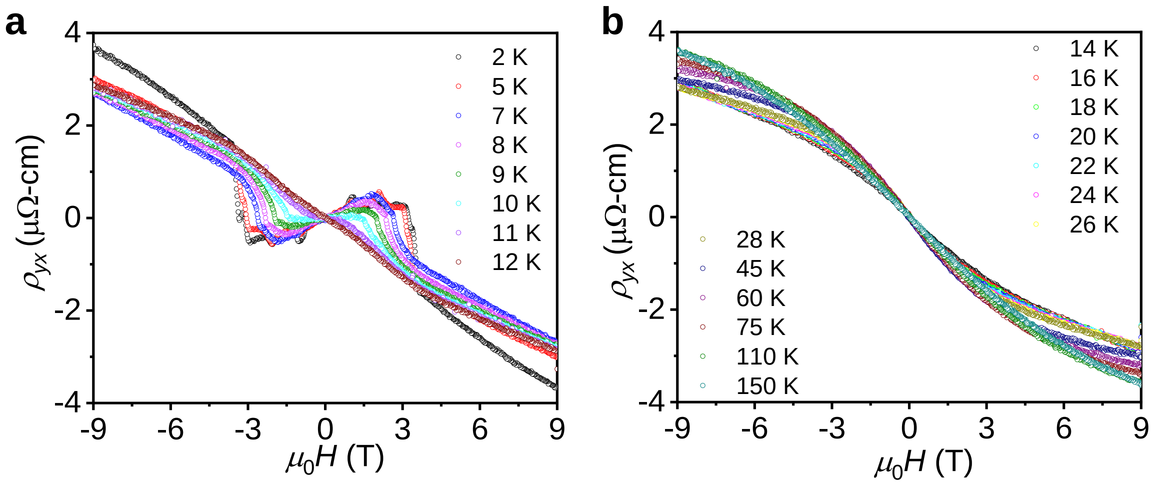

Fig. S5. Field dependent total Hall resistivity (ρ_{yx}) at different temperatures. Measurement configuration: *H* || *a* and *I* || *c* axis of HoAgGe crystal.

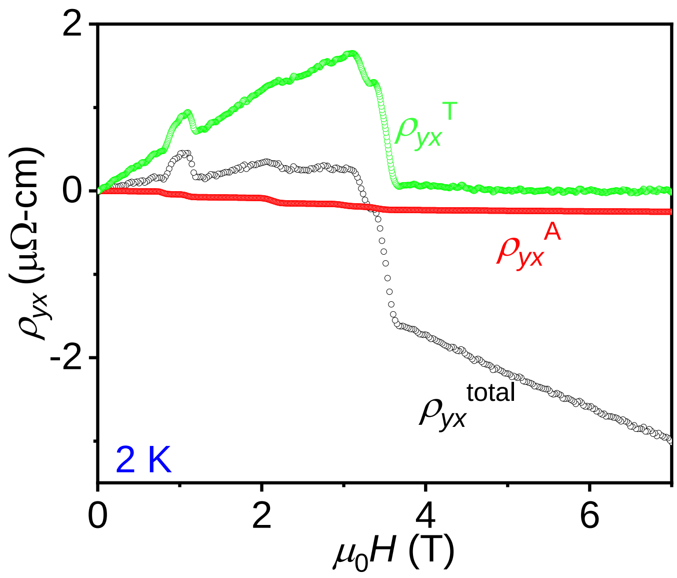

Fig. S6. Field dependent decoupling of total Hall resistivity, ρ_{yx}^{total} to the anomalous Hall resistivity, ρ_{yx}^{A} and the topological Hall resistivity, ρ_{yx}^{T} at 2 K.

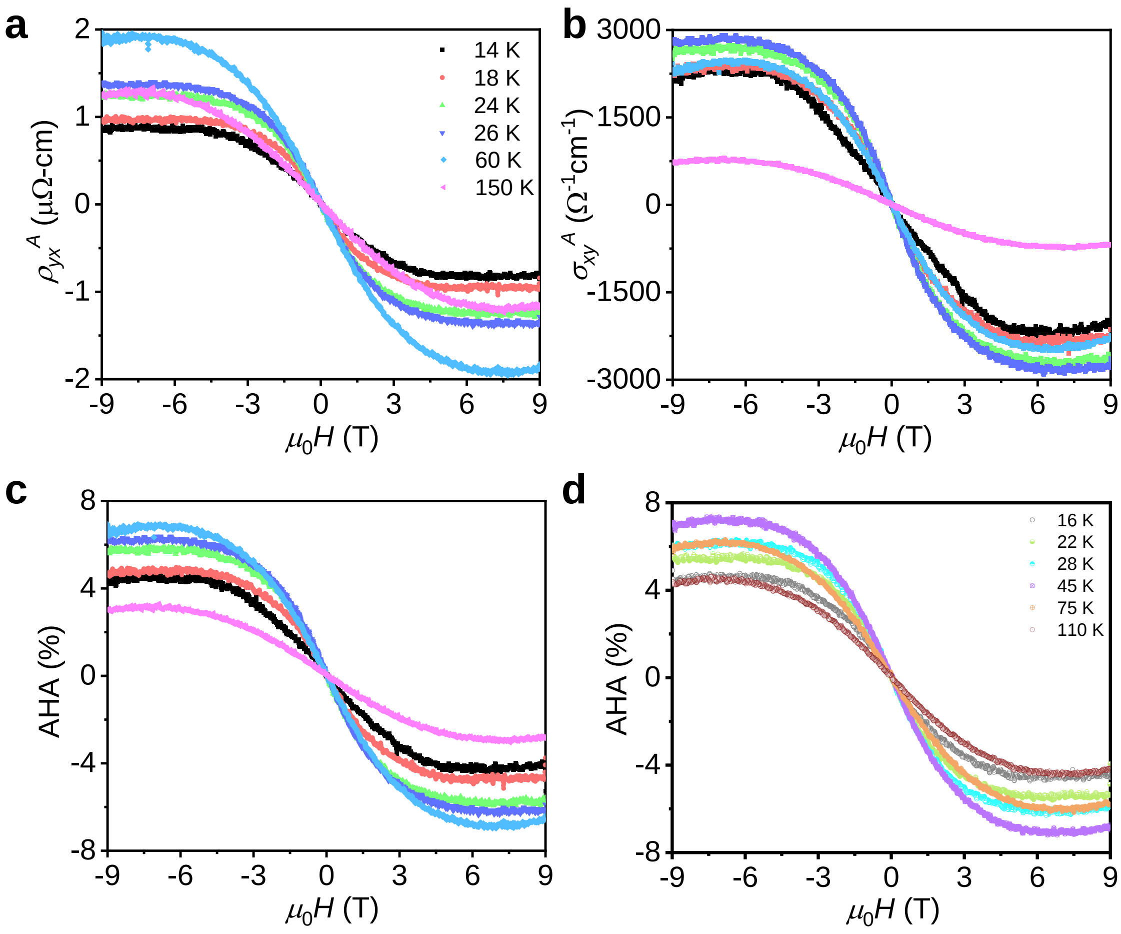

Fig. S7. Field-dependent **a.** anomalous Hall resistivity (ρ_{yx}^{A}), **b.** anomalous Hall conductivity, σ_{xy}^{A}, and **c and d.** anomalous Hall angle (AHA) of HoAgGe at different temperatures. Measurement configuration: *H* || *a* and *I* || *c* axis of HoAgGe crystal. In the main text, anomalous electrical transport data below and above (few temperatures) the Néel temperature are presented.

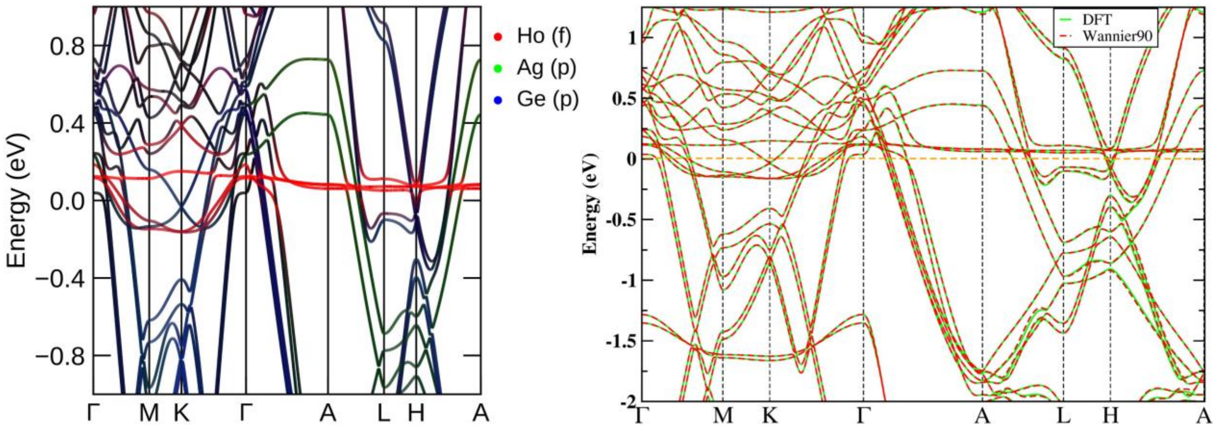

Fig. S8: Left panel: Site projected band structure of forced ferromagnetic (FM) ordered state in GGA+*U*+SOC showing the dominant orbital character around the Fermi energy. **Right panel:** Band structure in GGA+*U*+SOC for FM state with magnetic moment along the *c*-axis. Green lines: GGA+*U*+SOC first principles electronic bands. Red lines: Wannier-interpolated Ho-*s,d,f,* Ag-*s,p,* Ge-*p* band structure.

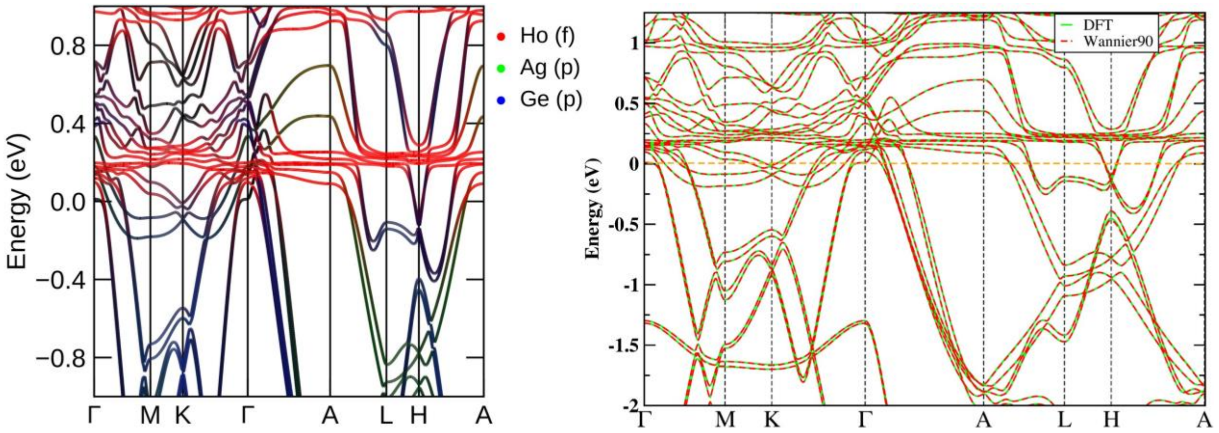

Fig. S9: Left panel: Site projected band structure of antiferromagnetically ordered (AFM) (2in-1out configuration) in GGA+*U*+SOC showing the dominant orbital character around the Fermi energy. **Right panel:** Band structure in GGA+*U*+SOC for AFM (2in-1out spin-ice configuration) state with magnetic moment along the z-axis. Green lines: GGA+*U*+SOC first principles electronic bands. Red lines: Wannier-interpolated Ho-*s,d,f,* Ag-*s,p,* Ge-*p* band structure.

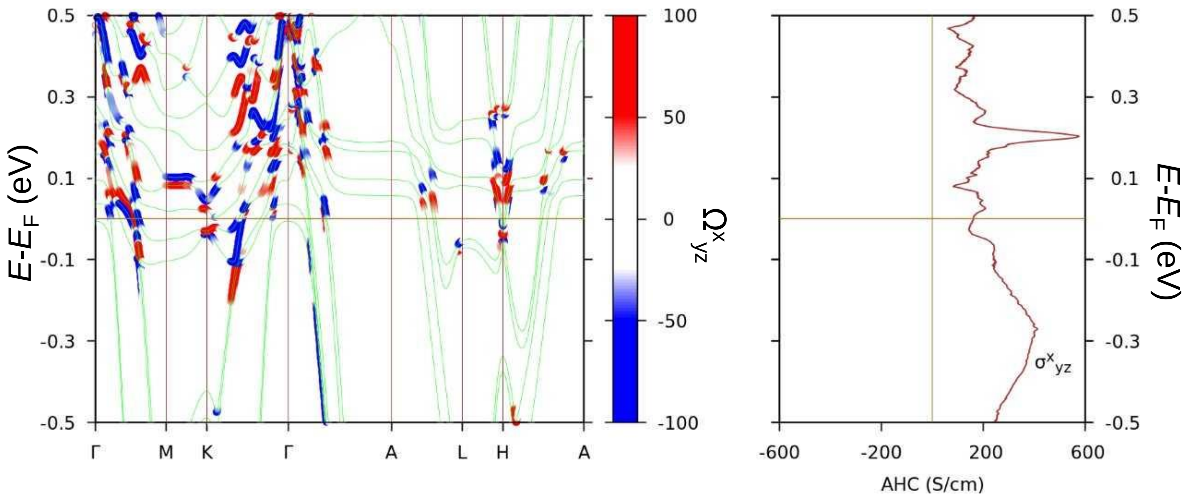

Fig. S10: Band structure of HoAgGe for the AFM (2in-1out configuration) state with magnetic moment along the *a*-axis and theoretically calculated Hall conductivity as a function of chemical potential. Red and blue colors indicate the Berry curvature (BC) contribution in the AFM state.

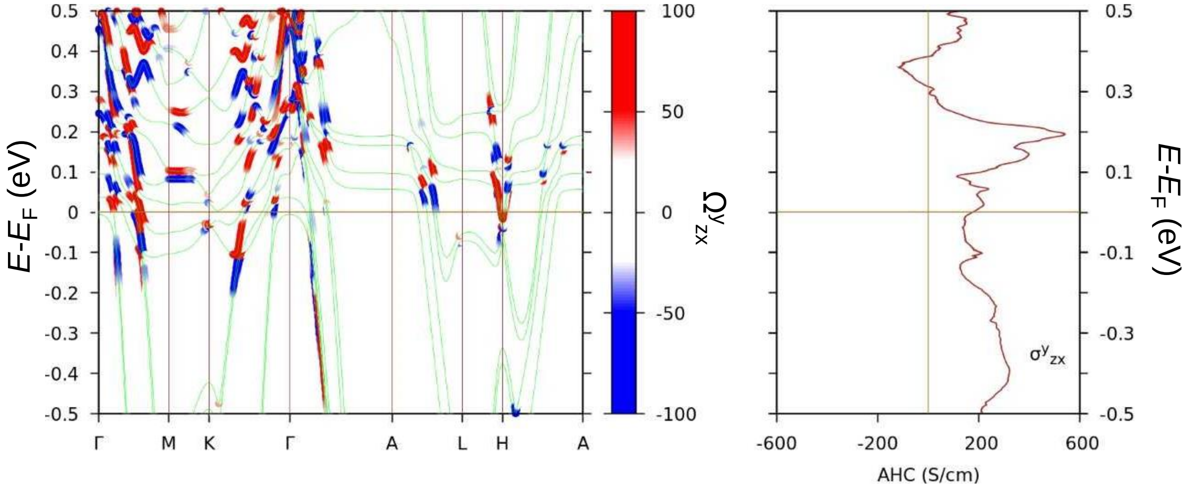

Fig. S11: Band structure of HoAgGe for the AFM (2in-1out configuration) state with magnetic moment along the *c*-axis and theoretically calculated Hall conductivity as a function of chemical potential. Red and blue colors indicate the BC contribution in the AFM state.

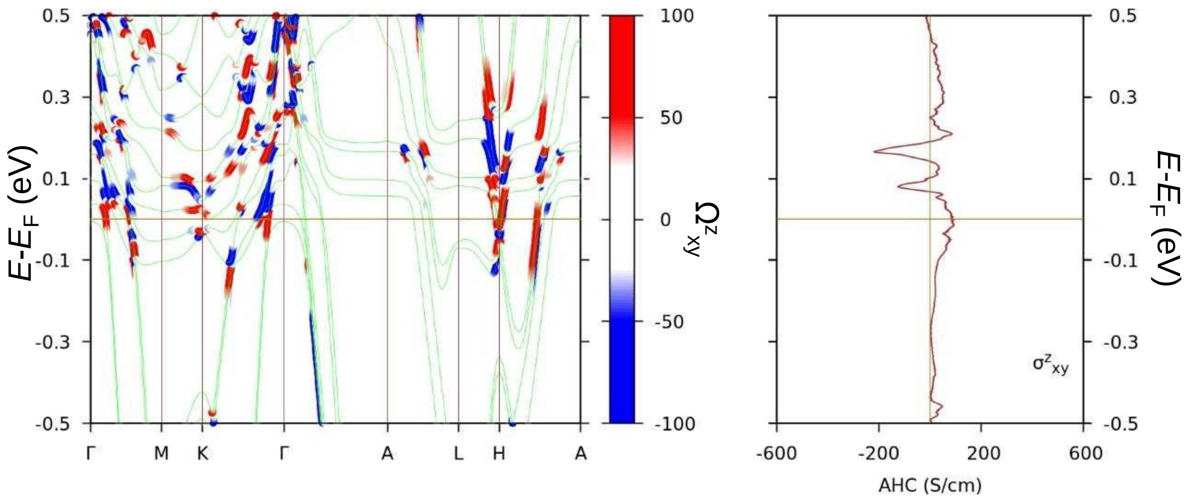

Fig. S12: Band structure of HoAgGe for the AFM (2in-1out configuration) state with magnetic moment along the *c*-axis and theoretically calculated Hall conductivity as a function of chemical potential. Red and blue colors indicate the BC contribution in the AFM state.
